# An Aptamer Sensor Based on Alendronic Acid-Modified Upconversion Nanoparticles Combined with Magnetic Separation for Rapid and Sensitive Detection of Thiamethoxam

**DOI:** 10.3390/foods14020182

**Published:** 2025-01-09

**Authors:** Qian Huang, Lu Han, Hui Ma, Weijie Lan, Kang Tu, Jing Peng, Jing Su, Leiqing Pan

**Affiliations:** 1 College of Food Science and Technology, Nanjing Agricultural University, Nanjing 210095, China; 2023108039@stu.njau.edu.cn (Q.H.); 2022208020@stu.njau.edu.cn (L.H.); 2022208019@stu.njau.edu.cn (H.M.); weijie.lan@njau.edu.cn (W.L.); kangtu@njau.edu.cn (K.T.); jpeng@njau.edu.cn (J.P.); 2 Huai’an Food and Drug Inspection Institute, Huai’an 223003, China

**Keywords:** thiamethoxam, upconversion nanoparticles, aptamer, biosensor, magnetic nanoparticles

## Abstract

The widespread use of thiamethoxam has led to pesticide residues that have sparked global concerns regarding ecological and human health risks. A pressing requirement exists for a detection method that is both swift and sensitive. Herein, we introduced an innovative fluorescence biosensor constructed from alendronic acid (ADA)-modified upconversion nanoparticles (UCNPs) linked with magnetic nanoparticles (MNPs) via aptamer recognition for the detection of thiamethoxam. Through base pairing, thiamethoxam-specific aptamer-functionalized MNPs (apt-MNPs) were integrated with complementary DNA-functionalized UCNPs (cDNA-UCNPs) to create the MNPs@UCNPs fluorescence biosensor. Thiamethoxam specifically attached to apt-MNPs, leading to their separation from cDNA-UCNPs, which in turn led to a reduction in fluorescence intensity at 544 nm following separation by an external magnetic field. The change in fluorescence intensity (ΔI) was directly correlated with the concentration of thiamethoxam, enabling the quantitative analysis of the pesticide. With optimized detection parameters, the biosensor was capable of quantifying thiamethoxam within a concentration span of 0.4–102.4 ng·mL^−1^, and it achieved a detection limit as minute as 0.08 ng·mL^−1^. Moreover, leveraging the swift magnetic concentration properties of MNPs, the assay duration could be abbreviated to 25 min. The research exhibited a swift and precise sensing platform that yielded promising results in samples of cucumber, cabbage, and apple.

## 1. Introduction

Neonicotinoid pesticides are a class of insecticides that have been developed since the 1980s [1]. Because of their unique mechanism of action and high efficiency and broad-spectrum insecticidal effect, neonicotinoid pesticides have emerged as the most rapidly expanding category of insecticides. [2,3,4]. As a second-generation neonicotinoid, thiamethoxam has a novel structure with good gastrotoxicity, contact toxicity, and endotoxic activity, making it one of the most profitable and widely used neonicotinoids [5]. However, the safety of thiamethoxam and its metabolites are increasingly being called into question due to their ongoing accumulation in plant tissues and soil [6]. Its toxic legacy in crops damages the biological environment and jeopardizes human health [7,8]. The U.S. Environmental Protection Agency released a final biological assessment in June 2022 for three neonicotinoid insecticides (thiamethoxam, imidacloprid, and acetamiprid), which have the potential to adversely affect 77% of species and 81% of critical habitats and harm a vast majority of endangered species (fish, birds, and mammals) [9,10,11]. Hence, a straightforward, swift, and dependable method for detecting thiamethoxam is crucial to safeguard against its adverse impacts.

The detection methods of thiamethoxam include gas chromatography, liquid chromatography, liquid chromatography tandem mass spectrometry, gas chromatography tandem mass spectrometry, and other instrumental methods [12,13,14,15]. These methods offer the benefits of high sensitivity, robust resistance to interference, and precise quantification, but the sample pretreatment is complicated, the detection cost is high, and it cannot meet the demand of the rapid detection of large-volume samples [16,17]. In contrast to electrochemical sensors, fluorescence sensors operate without the need for current measurement, rendering them less prone to electromagnetic disturbances. Furthermore, the absence of a requirement for reference electrodes and electrolytes in fluorescence sensors not only prevents measurement inaccuracies stemming from electrolyte depletion but also enhances the longevity of the sensors [18,19]. The fluorescence properties of traditional fluorescent substances, including organic fluorescent dyes and quantum dots (QDs), are susceptible to degradation during the detection process, thus limiting their application in fluorescence sensors [20]. UCNPs have emerged as unique fluorescent materials that can stepwise convert low-energy photons (near-infrared excitation) into high-energy photons (visible emission) [21,22]. They possess superior optical stability, extended fluorescence lifetimes, reduced toxicity, and are more resistant to photobleaching [23,24,25]. These characteristics of UCNPs provide them with great advantages in the construction of fluorescence sensors [26,27]. Simultaneously, aptamers partially address the challenges related to antibody size, stability, and expense that immunosensors encounter, while also leveraging their unique benefits, such as a brief screening period, minimal working concentrations, robust stability, economic viability, and convenient storage. Consequently, the fluorescence aptamer sensor, which integrates the strengths of both fluorescence and aptamer technologies, has emerged as an exceptionally valuable and significant tool for the detection of pesticide residues [28]. Moreover, MNPs characterized by excellent water dispersibility and the capacity for rapid separation and enrichment are extensively utilized in immunoassays, cell separation, and biomedical applications [29,30,31]. He et al. utilized MNPs to modify graphene oxide nanosheets (GOs), which were then combined with UCNPs for viral DNA detection [32]. Huang et al. cleverly combined MNPs antibody probes with SERS signal amplification technology, exhibiting superparamagnetism and highly specific recognition, for the detection of procalcitonin [33]. It was found that these assays incorporating MNPs have good sensitivity and can be analyzed to the target detectors in a short time due to the magnetic separation property. Therefore, alendronic acid-modified UCNPs can be considered for the construction of fluorescence biosensors based on thiamethoxam aptamers by combining alendronic acid-modified UCNPs with MNPs.

**Scheme 1:**
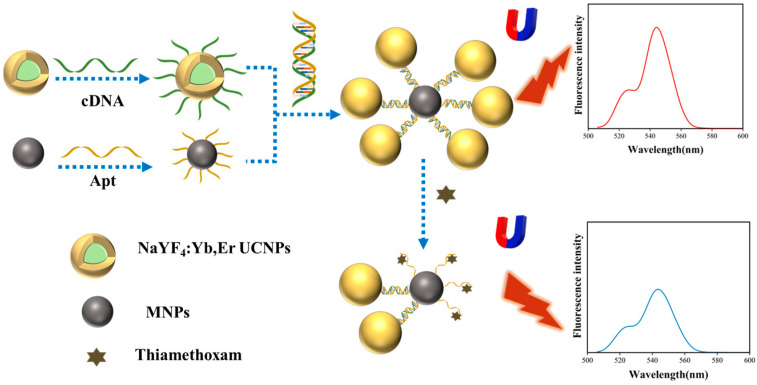
Schematic illustration of the strategy to form the MNPs@UCNPs fluorescence biosensor for thiamethoxam detection.

Herein, we fabricated a novel upconversion fluorescence biosensor based on label-free aptamer for the quantitative detection of thiamethoxam pesticide residues. Scheme 1 depicts the sensing mechanism of the proposed method. Employing the magnetic enrichment capacity of MNPs, MNPs@UCNPs undergoes magnetic separation under the action of an external magnetic field, which leads to changes in fluorescence intensity. Thiamethoxam selectively binds to the aptamer, disrupting the linkage between MNPs and UCNPs, leading to the detachment of UCNPs. The fluorescence intensity in the system to be weakened by magnetic separation and then resuspension. Subsequent magnetic separation and resuspension cause a decrease in the fluorescence intensity. Consequently, the specific interaction between the aptamer and varying concentrations of thiamethoxam was exploited to achieve a linear decrease in the system’s fluorescence intensity with increasing thiamethoxam concentration. This method can be utilized for a straightforward and effective approach to detect thiamethoxam in cucumber, cabbage, and apple samples.

## 2. Materials and Methods

### 2.1. Materials

Rare earth nitrates were obtained from Macklin Biochemical Technology Co., Ltd. (Shanghai, China). The following chemicals were procured from Sigma Aldrich Co., Ltd. (Shanghai, China): 1-octadecene, oleic acid (OA), alendronic acid (ADA), chloroform, hydrochloric acid, ammonium fluoride, sodium hydroxide, ferric chloride, 1,6-hexanediamine, and anhydrous sodium acetate. Acetamiprid (99%) and other pesticide standards were purchased from Sango Biotechnology Co., Ltd. (Shanghai, China). The thiamethoxam-specific aptamer and the complementary strand were supplied by Sango Biotechnology Co., Ltd. (Shanghai, China) with the sequence 5′-biotin-TAT GTT CTT AAC TGG TCG TCC TGT GAG CCG ATC ACT AGA TAA TTA GGA T -3′ (apt) and 5′-NH_2_-A TCC TAA TTA TCT AGT GAT CGG CTC ACA GGA CGA CCA GTT AAG AAC ATA -3′ (cDNA) [34].

### 2.2. Apparatus

UCNPs and MNPs were visualized under Talos L120C TEM (Thermo Fisher Scientific, Waltham, MA, USA). The surface groups of OA-UCNPs, ADA-UCNPs, and MNPs were analyzed with an FT-IR spectrometer (Perkin Elmer, Waltham, MA, USA) using KBr pellets. The crystal structures were studied with an X-ray diffractometer (Bruker D8 Advance, North Billerica, MA, USA). The information on the magnetization curve of MNPs was provided by a Vibrating Sample Magnetometer (LakeShore 8600, Westerville, OH, USA). The upconversion fluorescence spectra were recorded using the in-studio upconversion fluorescence detection system.

### 2.3. UCNPs Synthesis and Surface Modifications

NaYF_4_:Yb/Er UCNPs, doped with rare earth metals, were fabricated using a high-temperature thermal decomposition technique as described in the literature [35,36]. YCl_3_·6H_2_O (0.1164 g), ErCl_3_·6H_2_O (0.0060 g), YbCl_3_·6H_2_O (0.0620 g), and GdCl_3_·6H_2_O (0.0892 g) were dissolved in methanol (2 mL). The mixture was then mixed with 6 mL of oleic acid and 14 mL of 1-octadecene. Under the protection of nitrogen, the mixture was heated to 160 °C, followed by continuous magnetic stirring for 30 min until completely dissolved. Then, a methanol solution of NaOH and NH_4_F was added and the mixture was reacted in a water bath at 50 °C for 30 min, followed by complete evaporation at 70 °C. After the complete removal of methanol, the mixture was rapidly heated to 300 °C and reacted for 1 h and then cooled to room temperature. The synthesized products, i.e., OA-UCNPs, were cleaned using a mixture of cyclohexane and ethanol and placed in an oven at 60 °C for 8 h.

In order to construct an upconversion fluorescence biosensor, the water-soluble modification of OA-UCNPs is required. Therefore, in this experiment, the OA-UCNPs were modified with alendronic acid [37]. A quantity of 25.0 mg of alendronic acid was dissolved in 3 mL of deionized water, to which 50 mg of OA-UCNPs, 5 mL of CHCl_3_, and 2 mL of ethanol were subsequently added. The combined solution was then subjected to sonication for a duration of 30 min. The pH of this mixture was adjusted to a range of 2–3 using HCL (0.1 M), and the mixture was stirred magnetically for 1 h to facilitate the reaction. Following the reaction, the resultant products were cleansed with a blend of ethanol and water. The alendronic acid-modified UCNPs were then dried in an oven at 60 °C for 8 h after the washing process.

### 2.4. Synthesis of Amine-Functionalized Fe_3_O_4_ Magnetic Nanoparticles

The amine-functionalized MNPs were synthesized via a literature-reported one-step method [38]. The synthesis process was as follows. Initially, 2.0 g of anhydrous sodium acetate, 6.4 g of 1,6-hexanediamine, and 1.0 g of ferric chloride hexahydrate were precisely measured and mixed into 45 mL of ethylene glycol, after which the mixture was placed in a 50 °C water bath. After the solution became clear and transparent, it was transferred to a polytetrafluoroethylene (PTFE) liner and reacted in an oven at 198 °C for 6 h. Once cooled to 25 °C, the MNPs were magnetically isolated and rinsed with deionized water 2–3 times to yield amine-functionalized MNPs, which were then dried at 60 °C overnight.

### 2.5. Synthesis of Aptamer and cDNA-Conjugated Nanoparticles

Alendronic acid-modified UCNPs were attached to the complementary chains by the classical glutaraldehyde method. UCNPs, accurate to 4 mg, were weighed out and combined with 2 mL of PBS buffer (0.01 M, pH = 7.2) before being sonicated for 30 min. Then, 0.6 mL of glutaraldehyde solution was introduced, and the mixture was magnetically stirred for 2 h. The activated UCNPs were then rinsed 2–3 times with PBS buffer. Following this, 30 μL of 100 μM cDNA was added to the mixture, which was placed on a shaker at 37 °C overnight. The solution was subjected to centrifugation at 8000 rpm for 3 min, the supernatant was removed, and the pellet was washed 2–3 times with PBS buffer. The cleaned cDNA-UCNPs were dispersed into 10 mL of PBS buffer and stored in a refrigerator at 4 °C [39]. In addition to isolation of MNPs from the mixture by streptavidin activation and magnetic separation, apt-MNPs were prepared by using a method similar to that used for cDNA-UCNPs, with the specific preparation steps provided in the Supplementary Materials.

### 2.6. Analytical Procedure

To monitor thiamethoxam using the method we developed, 50 μL of cDNA-UCNPs (0.4 mg/mL) and 140 μL of apt-MNPs (2 mg/mL) were incubated with 310 μL of PBS buffer (0.01 M, pH = 7.2) in a 1 mL centrifuge tube for 30 min at 25 °C to obtain the complexes (MNPs@UCNPs). Unbound cDNA-UCNPs and apt-MNPs were washed using PBS buffer with the aid of a magnetic separator. The washed mixture was redissolved in 250 μL of PBS buffer, followed by the addition of 250 μL of thiamethoxam standard solution. After 30 min of interaction, the complexes were washed gently with PBS buffer several times, then resuspended in 500 μL of PBS buffer, and the fluorescence intensity was recorded at an excitation/emission wavelength of 980/544 nm.

### 2.7. Method Specificity

To assess the specificity of the developed method, the MNPs@UCNPs fluorescence biosensor was used to analyze 2,4-D, dinotefuran, deltamethrin, imidaclothiz, acetamiprid, imidacloprid, and thiamethoxam at a concentration of 6.4 ng·mL^−1^. The fluorescence intensities of various pesticides were recorded. The fluorescence intensity in the solution system before and after the presence of the target detector was recorded as I_0_ and I, respectively, and the difference between the two was recorded as ΔI (ΔI = I_0_ − I).

### 2.8. Preparation and Detection of Authentic Samples

To evaluate the usefulness and feasibility of the constructed MNPs@UCNPs fluorescence biosensor, cucumber, cabbage, and apple were selected for testing. Cucumbers, cabbages, and apples were purchased from local supermarkets in Nanjing. For the analysis of cucumber samples, fresh tissue (10 g) was chopped and homogenized in a fruit and vegetable grinder. The resulting cucumber juice was centrifuged at 12,000 rpm for 8 min and then purified using a 0.22 μm syringe membrane filter. Finally, different concentrations of thiamethoxam standard solutions were added for assay, each concentration was determined three times, and the recoveries and relative standard deviations were calculated. The detection procedure is the same as mentioned in the analytical procedure section. Cabbages and apples were treated in the same way as cucumbers.

## 3. Results and Discussion

### 3.1. Characterization of MNPs@UCNPs Fluorescence Biosensor

**Figure 1:**
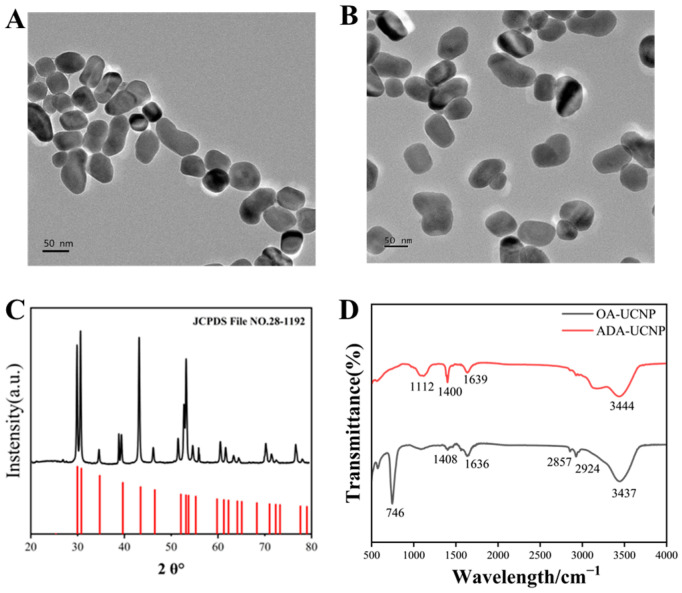
Characterization of UCNPs: (**A**) TEM image of OA-UCNPs, (**B**) TEM image of ADA-UCNPs, (**C**) XRD of UCNPs, and (**D**) FT-IR spectra of OA-UCNPs and ADA-UCNPs.

The synthesized nanoparticles are characterized by a range of properties including morphology, size, crystallinity, and surface groups. Figure 1A,B shows TEM images of UCNPs prior to and following modification with alendronic acid. The UCNPs before modification showed an elliptical shape. The size and morphology of the UCNPs remained largely unchanged following modification with alendronic acid. The composition and crystalline structure of the UCNPs were determined by XRD and matched against the Na(Y_0_._57_Yb_0_._39_Er_0_._04_) F_4_ standard card (JCPDS file No. 28-1192) (Figure 1C). The distinctive peaks observed matched those of the UCNPs standard cards, signifying that the synthesized UCNPs possess a well-defined crystalline structure. The surface functional groups of OA-UCNPs and ADA-UCNPs were identified through FT-IR spectroscopy, with the resulting spectra depicted in Figure 1D. The vibrational peaks at 1408 cm^−1^ and 1636 cm^−1^ were attributed to the symmetric and asymmetric stretching vibrations of the carboxyl group (-COO-) in the OA. Meanwhile, the peaks at 746 cm^−1^, 2857 cm^−1^, and 2924 cm^−1^ were associated with the in-plane deformation wobble and symmetric and asymmetric stretching vibrations, respectively, of the methyl group (-CH_2_-) in the lengthy alkyl chain of OA [40]. Following the ADA modification, a noticeable reduction in the aforementioned peak intensities was detected, signifying the effective elimination of the oleic acid layer from the UCNPs’ surface. The emergence of peaks at 1112 cm^−1^ and 1639 cm^−1^ were attributed to the P=O stretching vibration and the amino (NH_2_) deformation vibration, respectively.

**Figure 2:**
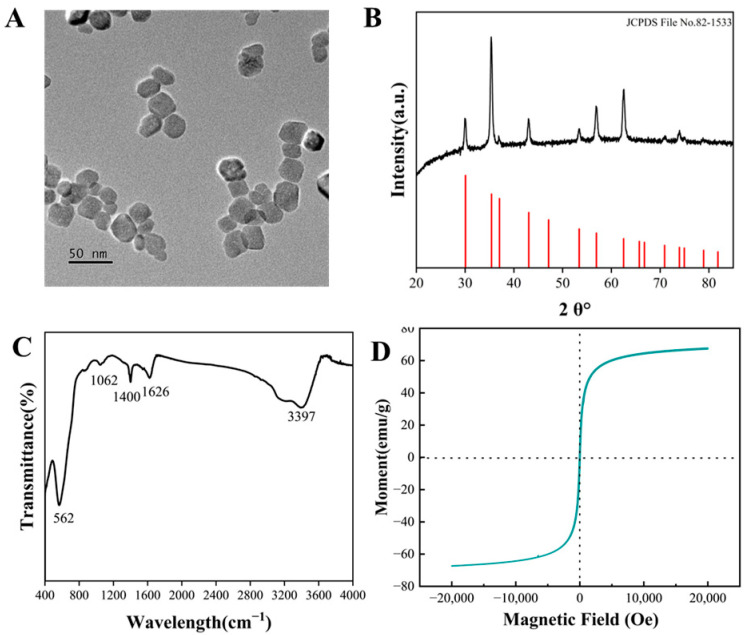
Characterization of MNPs: (**A**) TEM image of MNPs, (**B**) XRD of MNPs, (**C**) FT-IR spectrum of MNPs, and (**D**) magnetization curve of MNPs.

Figure 2 demonstrates the characterization results of the MNPs. The average diameter of the synthesized MNPs was around 25 nm, as shown in Figure 2A. The XRD and FT-IR characterization results of the MNPs are shown in Figure 2B,C. The XRD pattern corresponded with the standard card for Fe_3_O_4_ as documented in the JCPDS file no. 82-1533. The peak observed at 568 cm^−1^ was associated with the Fe-O stretching vibration. Meanwhile, the peaks at 3397 cm^−1^ and 1626 cm^−1^ were due to the N-H stretching and deformation vibrations, respectively. These findings suggested that amino groups are present on the surface of the MNPs. Figure 2D illustrates the magnetization curve of MNPs measured at room temperature, featuring a saturation magnetization of 67.8 emu/g. The curve is characteristic of an S-shape with negligible remanence and coercivity, suggesting the absence of hysteresis. This confirms the superparamagnetic nature of the synthesized MNPs.

The MNPs@UCNPs fluorescence biosensor constructed in this study was composed of signal carrier UCNPs, magnetic separation medium MNPs, and biorecognition element aptamers. UCNPs provided fluorescent signals to the sensor, MNPs rapidly separated and enriched the target pesticide molecules, and aptamers preferentially captured the target pesticide molecules. The amino group carried on the surface of alendronic acid-modified UCNPs interacted with the amino group modified at the cDNA terminus and achieved the covalent binding of cDNA driven by chemical bonding covalent binding forces. MNPs were activated by streptavidin, which interacted with biotin modified at the end of the aptamer to achieve covalent binding of the aptamer. Figure S1 demonstrates UV spectral images of the UV spectra of apt-UCNPs and cDNA-MNPs. The results show that there is an absorption peak at 260 nm, which is because aptamer and cDNA are essentially nucleic acids, and they have UV absorption peaks at 260 nm. This proves the successful combination of aptamer and cDNA with nanomaterials. In addition, we obtained upconversion fluorescence spectrograms under different components. As shown in Figure S2, the fluorescence intensity of the solution at 544 nm remained almost the same after incubating the cDNA-UCNPs with apt-MNPs for sufficient time. This indicates that apt-MNPs and cDNA-UCNPs have been linked together by base interactions, and after magnetic separation and then resuspension, the fluorescence intensity in the system is almost unchanged. However, after adding thiamethoxam to the MNP@UCNPs solution system after incubation, the fluorescence intensity at 544 nm of the solution underwent a significant attenuation. This is because thiamethoxam preferentially captures apt-MNPs, which causes the cDNA-UCNPs to fall off, and the fluorescence intensity in the solution obtained by magnetic separation and then resuspension decreases. Therefore, the principle of our assay is feasible.

### 3.2. Optimization of Detection Conditions

To enhance the sensitivity of the MNPs@UCNPs fluorescence biosensor, optimization was carried out on three assay parameters: the volume of apt-MNPs, the incubation time of cDNA-UCNPs with apt-MNPs, and the response time of MNPs@UCNPs with thiamethoxam.

**Figure 3:**
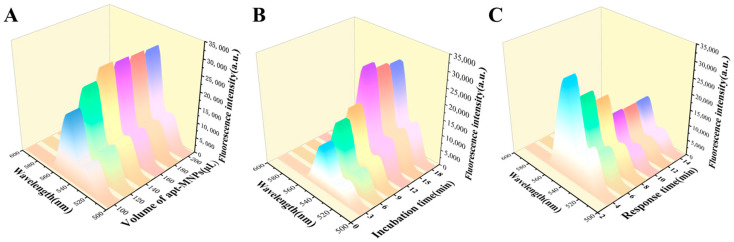
Optimization of (**A**) volume of apt-MNPs, (**B**) incubation time, and (**C**) response time.

In the sensing system, MNPs were used as a carrier for aptamers and as a separation medium. If an insufficient amount was added, it would affect the adequate binding of aptamer to cDNA, resulting in weak fluorescence signals and affecting the detection results. If too much were added, the color of the reaction system would deepen, which may cause background interference and affect the fluorescence value, leading to waste. With the fixed addition amount of cDNA-UCNPs, the trend of fluorescence intensity at 544 nm in the system gradually leveled off when the addition amount of apt-MNPs reached 140 μL (Figure 3A). The optimal additional amount of apt-MNPs was determined to be 140 μL to fully ensure the fluorescence signal intensity and not to cause material waste. The fluorescence intensity measured at 544 nm reaches a maximum when the incubation time reaches 12 min (Figure 3B). As the incubation time continues to increase, the fluorescence intensity can be basically maintained. Upon the completion of the incubation, due to the addition of the target detector thiamethoxam to the system, the apt-MNPs will preferentially bind thiamethoxam, resulting in the shedding of the cDNA-UCNPs, and thus a decrease in the fluorescence intensity at 544 nm is observed (Figure 3C). When the reaction time of thiamethoxam with MNPs@UCNPs reached 10 min, the trend of fluorescence intensity at 544 nm remained essentially unchanged. Hence, to enhance the detection efficiency, it was established that the ideal incubation time was 12 min, while the optimal response time was 10 min.

### 3.3. Analytical Performance of MNPs@UCNPs Fluorescence Biosensor

**Figure 4:**
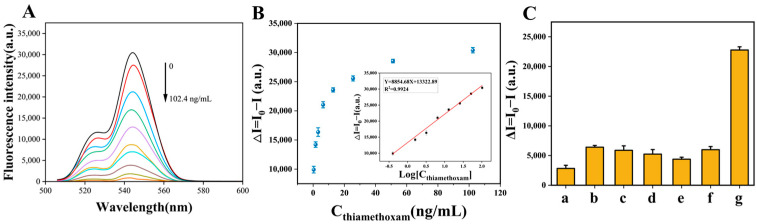
(**A**) Upconversion fluorescence spectrum of thiamethoxam at different concentrations. (**B**) Standard curve of thiamethoxam. (**C**) Specificity evaluation of the MNPs@UCNPs fluorescence biosensor for thiamethoxam (a: 2,4-D; b: dinotefuran; c: deltamethrin; d: imidaclothiz; e: acetamiprid; f: imidacloprid; g: thiamethoxam). The error bars represent the standard deviations based on three independent measurements.

A series of thiamethoxam standard solutions (0.4–102.4 ng·mL^−1^) with concentration gradients were prepared and the fluorescence intensity values of the detection system at 544 nm were recorded under the optimized reaction conditions. The standard curve for the MNPs@UCNPs fluorescence biosensor was created by plotting the change in fluorescence intensity of the detection system. This change is denoted as ΔI, where ΔI is calculated as I_0_ minus I. Here, I_0_ represents the fluorescence intensity at 544 nm when thiamethoxam is absent from the detection system, while I represents the intensity in the presence of thiamethoxam. The value of fluorescence intensity in the reaction system decreased with increasing thiamethoxam concentration (Figure 4A). The linear regression equation was y = 8854.68x + 13322.89, R^2^ = 0.9924 (x represents the logarithmic value of thiamethoxam concentration and y represents ΔI). The limit of detection (LOD, F_0_-3SD) and detection range were 0.08 ng/mL and 0.4 to 102.4 ng/mL, respectively (Figure 4B). Table 1 shows a comparison of the performance of previously reported detection methods against thiamethoxam and the fluorescent biosensor invented in this study. The multicolor upconversion fluorescence immunoassay (UCFIA) exhibited a sensitivity comparable to MNPs@UCNPs biosensor invented in this study. However, it had a limited detection range and extended detection duration. The sensitivities for the other reported techniques were inferior to the MNPs@UCNPs biosensor, and they also required more time for detection than our proposed method, which confirmed the ideal performance of the sensor in this work.

### 3.4. Specificity Analysis

To access the specificity of the MNPs@UCNPs biosensors, the same concentrations of 2,4-D, dinotefuran, deltamethrin, imidaclothiz, acetamiprid, imidacloprid, and thiamethoxam were detected by this method. As shown in Figure 4C, the change in fluorescence intensity was significant in the presence of thiamethoxam, while the change in fluorescence intensity caused by the other pesticide molecules was almost negligible. The findings indicated that the method exhibits high specificity for thiamethoxam.

### 3.5. Determination of Thiamethoxam in Authentic Samples

To assess the sensitivity and suitability of the biosensor, cucumber, cabbage, and apple were selected as authentic samples for thiamethoxam detection. The average recoveries of these samples at concentrations of 0.5, 2, and 10 ng·mL^−1^ ranged from 94.67% to 103.65% in cucumber, 82.67% to 106.50% in cabbage, and 93.33 to 109.33% in apple, with corresponding relative standard deviations (RSDs) of 3.73–6.97%, 3.99–6.24%, and 5.65–8.55%, respectively (Table 2). Inter-batch RSDs ranged from 3.98 to 10.68%, indicating the good reproducibility of the batches. According to the guideline on pesticide residue trials of China (NY/T 788–2018), average recoveries are required to range from 70% to 110% and the RSDs should be no more than 15%. The precision and accuracy of this sensor meet these requirements. Meanwhile, the current GB 2763-2022 in China stipulates that the maximum residue limits (MRLs) of thiamethoxam in cucumber, cabbage, and apple are 0.5, 0.2, and 0.3 mg·kg^−1^, respectively. The results showed that the MNPs@UCNPs fluorescence biosensor can meet the requirements for the quantitative detection of thiamethoxam in cucumber, cabbage, and apple samples. Therefore, the sensor has satisfactory accuracy and applicability in the analysis of real samples.

## 4. Conclusions

This investigation developed a fluorescence biosensor by integrating ADA-UCNPs with MNPs and incorporating an aptamer as the recognition component for the precise detection of thiamethoxam. The biosensor benefited from the excellent biocompatibility of ADA-UCNPs and their distinctive emission peak at 544 nm, which allowed for a remarkable LOD of merely 0.08 ng·mL^−1^. Compared to most fluorescence biosensors, the fabricated fluorescence biosensor is more advantageous by the fact that UCNPs can be used for highly sensitive detection while MNPs are used for the rapid separation and enrichment of target pesticide molecules, and it can be used to simplify the detection principle by eliminating the need to involve in the design of fluorescence bursting mechanisms (e.g., FRET, IFE). Hence, it significantly improves the efficiency of the inspection process. The MNPs@UCNPs biosensor demonstrated unparalleled reliability and simplicity at trace levels. It is worth noting for future sensors that many interfering substances and multiple pesticide molecules often coexist in real food detection applications. In addition, compared with electrochemical sensors, for example, fluorescence aptamer sensors face challenges in continuous monitoring or field applications due to their susceptibility to interference from temperature, light, and other fluorescent substances. Therefore, the photothermal stability of UCNPs should be improved in the future, and miniaturized and portable broad-spectrum fluorescence sensors should be developed by integrating microfluidics and other technologies. 

## Figures and Tables

**Table 1:** Comparison between the MNPs@UCNPs fluorescence biosensor for thiamethoxam and other reported methods.

Method	Material	LOD (ng·mL^−1^)	Detection Range (ng·mL^−1^)	Detection Time (min)	Reference
Colorimetric aptasensor	AuNPs	0.49	1.46–43.76	40	[41]
Fluorescence biosensor	PQD, β-CD-SP	1.20	4.20–18.20	>30	[42]
Electrochemical detection	β-CD, GO	78.8	145.9–4600	30	[43]
UCFIA	UCNPs, MNPs	0.09	0.09–2.34	>60	[44]
SERS	β-CD, AgNPs	21.3	29.2–29,200	>60	[45]
UCNPs-MNPs biosensor	UCNPs, MNPs	0.08	0.4–104.2	25	This work

**Table 2:** Determination of thiamethoxam in authentic samples by the proposed method.

Samples	Additive Concentration (ng·mL^−1^)	Intra-Batch (n = 3)						Inter-Batch RSD (%) (n = 9)
		Batch 1		Batch 2		Batch 3		
		Average Recovery (%)	RSD (%)	Average Recovery (%)	RSD (%)	Average Recovery (%)	RSD (%)	
Cucumber	0.5	94.67	6.97	98.67	5.81	99.33	9.05	6.83
	5	96.33	9.54	103.65	4.21	101.04	4.77	7.14
	10	101.91	3.73	102.17	5.01	99.97	2.29	3.98
Cabbage	0.5	82.67	4.97	92.03	9.12	102.12	3.63	9.83
	2	102.33	3.99	102.83	4.51	106.50	2.13	4.08
	10	98.40	6.24	98.33	3.48	95.07	3.70	4.93
Apple	0.5	108.67	8.55	93.33	4.19	102.67	3.02	10.68
	2	109.33	7.32	94.51	3.02	107.17	6.72	8.85
	10	95.97	5.65	99.03	5.43	103.07	4.69	6.01

## Data Availability

The original contributions presented in the study are included in the article/Supplementary Materials, further inquiries can be directed to the corresponding author.

## Supplementary Materials

The following supporting information can be downloaded at: https://www.mdpi.com/article/10.3390/foods14020182/s1, Figure S1: UV spectra of apt-MNPs and cDNA-UCNPs; Figure S2: Upconversion fluorescence spectra of different components.
