# Supplementary material for: An Aptamer Sensor Based on Alendronic Acid-Modified Upconversion Nanoparticles Combined with Magnetic Separation for Rapid and Sensitive Detection of Thiamethoxam
Source: Foods. 2025 Jan 9;14(2):182. doi: 10.3390/foods14020182 (PMC11765007; doi:10.3390/foods14020182)
Supplement: Supplementary file 1 [file foods-14-00182-s001.zip › foods-3390966-supplementary.pdf]

SUPPORTING INFORMATION

An Aptamer Sensor Based on Alendronic Acid-Modified Upconversion Nanoparticles Combined with Magnetic Separation for Rapid and Sensitive Detection of Thiamethoxam

Qian Huang [1], Lu Han [1], Hui Ma [1], Weijie Lan [1], Kang Tu [1], Jing Peng [1], Jing Su [2,*] and Leiqing Pan [1,*]

[1] College of Food Science and Technology, Nanjing Agricultural University, Nanjing 210095, China; 2023108039@stu.njau.edu.cn (Q.H.); 2022208020@stu.njau.edu.cn (L.H.); 2022208019@stu.njau.edu.cn (H.M.); weijie.lan@njau.edu.cn (W.L.); kangtu@njau.edu.cn (K.T.); jpeng@njau.edu.cn (J.P.)

[2] Huai'an Food and Drug Inspection Institute, Huai'an 223003, China

* Correspondence: surpumpkin@163.com (J.S.); pan_leiqing@njau.edu.cn (L.P.)

1. Experimental Section

Synthesis of Aptamer -Conjugated Magnetic Nanoparticles

A quantity of 10 mg of MNPs was measured out and then dissolved in 5 mL of PBS buffer (0.01 M, pH=7.2). The mixture was subjected to sonication for a total of 30 min to ensure complete dispersion of the MNPs. To the solution, 2.5 ml of 25% glutaraldehyde was added rapidly and the reaction was carried out under magnetic stirring for 2 h. The MNPs were separated by an external magnetic field to obtain the MNPs and washed three times with PBS buffer (0.01 M, pH=7.2) to remove the unreacted glutaraldehyde. The product was redissolved in 5 ml of PBS buffer (0.01 M, pH=7.2), 100 μL of 1 mg/ml streptavidin was added, and the reaction was allowed to proceed overnight at room temperature. The reaction products were magnetically separated and washed three times with PBS buffer (0.01 M, pH=7.2) to remove unreacted streptavidin. Finally, the washed streptavidin-modified magnetic nanoparticles were dispersed into 5 ml of PBS buffer solution. After adding 30 μL of 100 μmol/L aptamer and shaking slowly at room temperature for 12 h, the supernatant was discarded after magnetic separation, and washed with PBS buffer (0.01 M, pH=7.2) for 3 times, the obtained apt-MNP were dispersed in 5 mL of PBS and stored at 4 °C, and stored at 4 ℃ for spare use.

2. Supplemental Figures and Tables

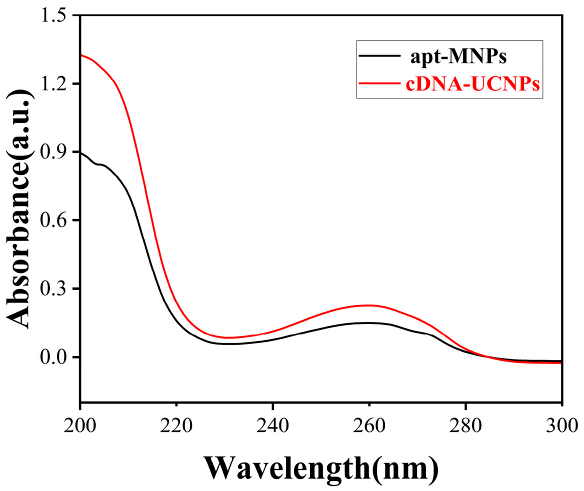

Figure S1. UV spectra of apt-MNPs and cDNA-UCNPs.

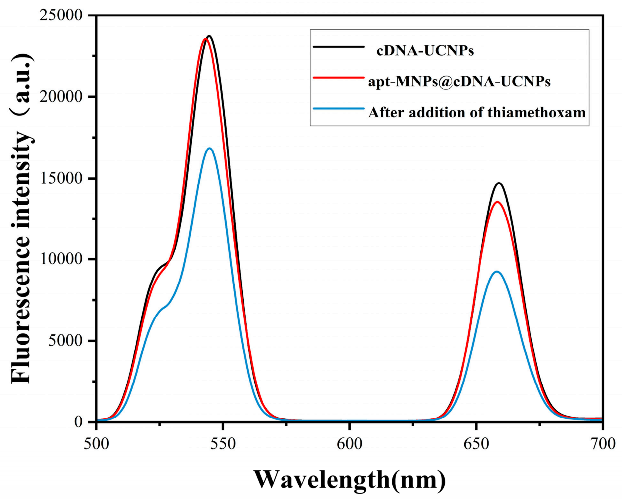

Figure S2. Upconversion fluorescence spectra of different components
